# Respiratory tract infection of fatal severe human bocavirus 1 in a 13-month-old child: A case report and literature review

**DOI:** 10.3389/fped.2022.949817

**Published:** 2022-12-20

**Authors:** Jing Liao, Zhongying Yang, Yu He, Jianhua Wei, Luo Ren, Enmei Liu, Na Zang

**Affiliations:** Department of Respiratory Medicine, Children's Hospital of Chongqing Medical University, Chongqing, China

**Keywords:** human bocavirus 1, severe infection, children, respiratory, *STAT1* variant

## Abstract

Human bocavirus 1 (HBoV1) belongs to the family Parvoviridae and it is acknowledged that HBoV1 is a respiratory pathogen. We report the case of a 13-month-old boy who presented with a cough, shortness of breath, and wheezing, and who eventually died of severe pneumonia and acute respiratory distress syndrome (ARDS). Metagenomics next-generation sequencing (mNGS) showed that HBoV1 was the only detected pathogen. The nasopharyngeal aspirate viral load was 2.08 × 10^10^ copies/ml and the serum viral load was 2.37 × 10^5^ copies/ml. The child was still oxygen deficient under mechanical ventilation. Chest imaging suggested diffuse lesions in both lungs, an injury caused by ARDS. In this case, the clinical symptoms and signs of the child, the high viral load, viremia, and the detection of mNGS in the tracheal aspirate all supported that HBoV1 could cause severe acute respiratory tract infection in children without other pathogen infections.

## Introduction

Human bocavirus 1 (HBoV1) was first discovered in respiratory secretions by Allander et al. in 2005 (1); later, three other bocaviruses (HBoV2, 3, and 4) were successively found in fecal samples (2). HBoV1, which belongs to the family of Parvoviridae and the genus of *Bocaparvovirus*, comprises a non-enveloped capsid with a single-stranded linear DNA and is the second known human parvovirus that can replicate autonomously after parvovirus B19 (3, 4). The clinical manifestations of HBoV1 infection are often atypical and similar to other respiratory virus infections, such as rhinitis, acute otitis media, pneumonia, bronchiolitis, and asthma exacerbation (5). The symptoms of HBoV1 infection are mild and self-limited and are easy to be ignored by clinicians. HBoV1 is often combined with other respiratory viruses, and the co-infection rate is as high as 75% (6, 7). The pathogenicity of HBoV1 was earlier considered controversial because HBoV1 DNA can persist for months in airway secretions following primary infection and can also be shed in the nasopharyngeal aspirates (NPAs) of asymptomatic healthy children. However, increasing evidence supports that HBoV1 is associated with respiratory symptoms without other pathogens detected and can even cause severe lower respiratory tract infections (8). A case of severe pneumonia, acute respiratory distress syndrome (ARDS), and respiratory failure brought on by HBoV1 infection was reported in our study, along with a review of the literature.

## Clinical data

A 13-month-old boy, the second child of consanguineous parents, was admitted to the intensive care unit (ICU) of the Children's Hospital of Chongqing Medical University in October 2018. He presented with a paroxysmal cough for 1 month, aggravated with shortness of breath and wheezing for 1 week. Regarding the patient’s family history, there was nothing unusual in the immune system. The child was delivered naturally at full term and has received his BCG vaccination. There was no history of feeding difficulties or repeated respiratory tract infections. He had not been exposed to toxic substances, gases, smog, or allergens.

A chest CT scan at a local hospital revealed a bilateral lung infection. The treatment of cefminox and interferon-α1b was not effective. The cyanosis was difficult to relieve. The patient was then admitted to our hospital with endotracheal intubation for further treatment. At admission, the boy had malnutrition with a normal body temperature and oxygen saturation of 94% under assisted ventilation (other vital signs were as follows: heart rate of 147 beats/min, the respiration rate of 30 beats/min, bodyweight of 5.5 kg, blood pressure of 77/50 mmHg). Nasal flaring, cyanosis around the lips, and an inspiratory triple concave sign were observed. Chest auscultation revealed bilateral coarse crackles.

**Figure 1:**
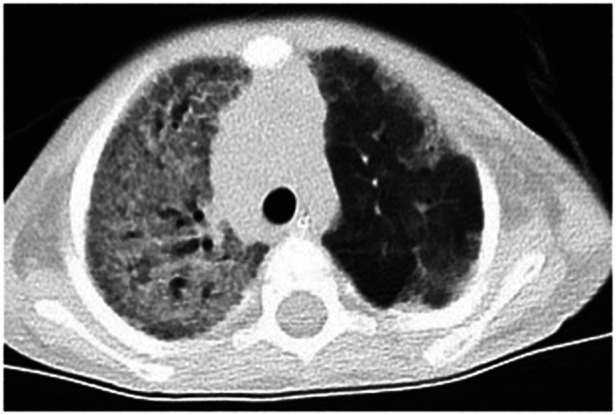
The chest-CT on the 14th day after hospitalization showed both lungs decreased translucency, extensive parenchymal opacities, and ground glass change. Some showed grid change and slightly dilated bronchus throughout the course.

Chest radiographs showed decreased translucency in both lungs and an extensively increased density shadow in the alveoli, showing ground-glass changes. Some showed a mesh change, a slightly dilated bronchus, and localized hyperinflation of the upper lobe of the left lung. Lung injury caused by a diffuse interstitial lesion of both lungs and ARDS were considered (Figure 1). The blood routine on admission showed that the white blood cell counts were elevated (18.91 × 10^9^/L) (reference value: 4.3–11.3 × 10^9^/L), neutrophils mainly, and a thrombocyte count of 490 × 10^9^/L (reference value: 100–453 × 10^9^/L), a hemoglobin concentration of 121 g/L (reference value: 118–156 × 10^9^ g/L), and a slightly elevated level of C-reactive protein (CRP) (9 mg/L) (reference value: <8 mg/L); other markers were within the reference ranges. After treatment with piperacillin-tazobactam for 4 days and meropenem for 6 days, the inflammatory indicators gradually decreased. However, on the 10th day of hospitalization, the white cell count and the neutrophil percentage increased again, and the level of CRP increased to 20 mg/L. The platelet count increased with the continuous decrease of red blood cells and hemoglobin (81–121 g/L), and the child had no bleeding tendencies. Amphotericin B, meropenem, and cefoperazone-sulbactam were administered as treatment, but the patient's condition did not improve. The level of CRP had no obvious change (18–20 mg/L), the white blood cell count continued to rise (12.25–18.45 × 10^9^/L), and the level of hemoglobin decreased to 77 g/L. Arterial blood gas analysis indicated type 2 respiratory failure (PO_2_53 mmHg, PCO_2_62 mmHg). After admission, the assisted ventilation was continued, and the patient still had persistent oxygenation disorders. The child had hypoproteinemia, increased lactate dehydrogenase, and a decrease in cholinesterase. Immunoglobulin levels (including IgM, IgG, and IgA) were in the normal range. The C3 complement was 0.43 g/L. Tumor markers were negative. Flow cytometry was used for the detection of T cell, B cell, NK cell and other lymphocyte subsets were determined, with no obvious abnormalities (CD4/CD8 2.37, CD3 + 1610.88/μl, CD3 + CD8 + 457.59/μl, CD3 + CD4 + 1084.62/μl, NK cell 53/μl). The TREC gene was detected by fluorescence probe PCR screening for severe combined immunodeficiency diseases. The result was >1,000 copies/μl (<10 copies/μl is considered to be severe combined immune deficiency, 10–1,000 copies/μl is considered to be a primary immune syndrome or normal, and >1,000 copies/μl is considered normal). The fungal D-glucan test, T-SPOT test, purified protein derivative test, *Mycoplasma pneumonia/Chlamydia* PCR, sputum bacterial and fungal culture, and double blood were negative. Pneumocystis was not detected in the next-generation sequencing (NGS) of bronchoalveolar lavage (BAL) fluid, and CT findings did not support pneumocystis Jiroveci pneumonia. All tests were negative for influenza virus A and B, respiratory syncytial, adenovirus, parainfluenza 1, 2, and 3, coronaviruses (including HKU-1, OC43, 229E, NL63), rhino/enterovirus, parechovirus, Hanta pulmonary syndrome, and fungi. The results of metagenomics NGS (mNGS) in serum and respiratory secretions suggested that HBoV had a high detection sequence of 112,786 reads and 100% gene coverage. The coverage of *Acinetobacter baumannii*, *Pseudomonas fluorescens*, and *Pseudomonas aeruginosa* were 0.889%, 0.058%, and 0.005%, respectively, and the reads were 370, 14, and 2, which were considered colonized bacteria. The mNGS was carried out by a core facility (Kindstar Global, Wuhan, China). HBoV was the only viral pathogen detected. No other pathogens were detected. Quantitative PCR (qPCR) showed that the viral load of NPA was 2.08 × 10^10^ copies/ml and the viral load of serum was 2.37 × 10^5^ copies/ml. Viral DNA and RNA were extracted from 200-µl aliquots of the NPA samples by the QIAamp MinElute Virus Spin kit (Qiagen, Hilden, Germany). The RNA was applied as the template for complementary DNA (cDNA) synthesis with the SuperScript III First-Strand Synthesis System (Invitrogen, California, USA). DNA and RNA extractions and cDNA products were used for the subsequent testing of respiratory viruses (9). HBoV1-specific primers were forward primer amplification of 5′-CCTATATAAGCTGCTGCACTTCCTG-3′ and reverse primer 5′-AAGCCATAGTAGACTCACCACAAG-3′ (10, 11). The plasmid amplified target fragment was cloned into the pMD19-T vector (TaKaRa Biotechonology, Dalian, China). The PCR process was performed exactly as described in the report (10), except for the AmpErase-UNG at 50°C. Each run included plasmid and negative controls. Standard precautions were taken throughout the PCR process to avoid cross-contamination. Negative controls and serial dilutions of the positive controls were included in every PCR assay. Therefore, HBoV was considered to be the pathogen in this case. Finally, the boy was diagnosed with severe pneumonia, ARDS, and diffuse pulmonary interstitial disease.

Due to the high suspicion of the possibility of respiratory system-related genetic diseases, complete exon sequencing (completed by Beijing Mygeno) was completed during hospitalization. It indicated that the patient had a heterozygous variant in the *STAT1* gene: C. 1127 + 10G > A, causing amino acid change splicing. After 16 days of treatment in the ICU, the patient's pulmonary symptoms were not alleviated, and the chest CT scan showed that the diffuse lesions in both lungs were still severe. The patient's family was required to give up treatment, and the patient died.

## Discussion

Since 2005, there has been increasing evidence supporting HBoV1 as an actual human pathogen that causes mild to severe respiratory infections. HBoV1 DNA is detectable in respiratory secretions in 2%–20% of children with acute respiratory tract infections (12). However, HBoV is not used to routinely detect respiratory viruses in clinical practice, which can easily lead to the neglect of clinicians and a missed diagnosis. However, increasing cases of severe and even fatal respiratory HBoV1 infections have been reported in recent years. In this study, the clinical characteristics, risk factors, methods of detection, and treatment of severe HBoV1 infection are discussed. Here, we present a case of severe HBoV1 infection and review the literature to provide a reference for its clinical diagnosis and treatment.

The major manifestations of severe HBoV1 infection were a respiratory failure or respiratory distress, fever, and wheezing. A total of 17 cases of HBoV1-related pediatric severe respiratory infection admitted to the ICU were collected from 10 pieces of literature (8, 13–21) (Table 1). Among these 17 children, fever and wheezing were the first symptoms in most cases. Specifically, the symptoms included respiratory failure or respiratory distress (*n* = 12), cough (*n* = 2), wheezing (*n* = 5), fever (*n* = 4), and diarrhea (*n* = 1). The diagnosis was respiratory failure (*n* = 5), ARDS (*n* = 4), bronchiolitis (*n* = 3), atelectasis (*n* = 2), and status asthma (*n* = 4), suggesting that severe HBoV infection often leads to respiratory failure and ARDS. Except for a 4-year-old child and two newborn patients, the children were aged between 6 months and 2 years. Chest imaging suggested interstitial infiltration and atelectasis. Specifically, it included atelectasis (*n* = 4), infiltration (*n* = 7), and hyperinflation (*n* = 1), which were consistent with our case. Our patient was 13 months old, and his first symptoms were a cough and fever, aggravated by shortness of breath, cyanosis, and hypoxemia. The chest CT scan suggested ground-glass changes, with diffuse lesions in both lungs. In the laboratory tests, with the increase in disease severity, the proportion of neutropenia and lymphocytopenia significantly increased. Procalcitonin and CRP obviously increased. In severe cases reported in the past, the levels of CRP and leukocytes were also slightly elevated. Among them, HBoV1 was the only pathogen detected. Jula et al. (12) reported that a tiny amount of HRV RNA was detected. However, the copy number of HBoV1 DNA was significant, so it was still highly correlated with HBoV1 infection.

**Table 1:** The reported severe respiratory tract infection cases admitted to ICU were caused by HBoV1.

Author	Country, year	Age	Male/female	Manifestation	Chest image	Sample	Method	Serology	Co-infection	Mechanical ventilation	Clinical diagnosis	Viremia	Viral loads in serum	Viral loads in NPA	Treatment	Outcome	Fundamental disease
Tabatabai et al. (11)	Germany, 2019	6 months	1/0	Respiratory failure	Bilateral opacities	Blood, tracheal secretions	PCR/serology	HBoV IgM/IgG	No	Yes	ARDS	Yes	2 × 10^3^	3.1 × 10^9^	Oxygen, antibiotics, NO	Death	Immunodeficient (NIN gene mutation, T-cell defect)
Moesker et al. (8)	The Netherlands, 2015	24 months	3/4	Respiratory failure, ECMO indication	–	NPA	PCR/NGS	–	No	5/7, 1/7ECMO	ARDS, LRTI, BHR/PSA, Severe atelectasis with ARTI	–	–	–	Oxygen, Supplemental	Survival	3Pulmonary disease, 1premature
Korner et al. (12)	Germany, 2011	8 months	0/1	Hypoxia, respiratory distress, wheezing, cough, and fever	Diffuse bilateral infiltrates and total atelectasis of the right upper lung lobe	Blood, NPA	PCR/serology	HBoV IgM/IgG	No	No	Severe obstructive bronchitis	Yes	5.8 × 10^3^	–	Oxygen, antibiotics	Survival	None
Eskola et al. (13)	Finland, 2017	9 months	1/0	Fever, wheezing, dyspnea	Interstitial infiltrates atelectasis of the right upper and lower lobes	Blood, tracheal aspirate	PCR/serology	HBoV IgM/IgG	No	Yes	Respiratory failure	Yes	1.7 × 10^3^	–	Oxygen, corticosteroids, antibiotics, NO	Survival	Bronchiolitis at 6 months
Ursic et al. (15)	Slovenia, 2011	20 months	0/1	Respiratory distress	Hyperinflation with an infiltrate in the left lower lung field	Blood, NPA, tracheal aspirate	PCR	–	No	Yes	Acute bronchiolitis, pneumomediastinum, interstitial emphysema, and acute respiratory failure	Yes	1.8 × 10^6^	8.6 × 10^9^	Oxygen, corticosteroids, antibiotics	Survival	Premature
Edner et al. (14)	Sweden, 2011	4 years	0/1	Fever, wheezing, dyspnea	Subcutaneous emphysema, pneumomediastinum, and left-sided pneumothorax	Blood, tracheal aspirate	PCR/serology	HBoV IgM	No	Yes and ECMO	ARDS	Yes	0.45 × 10^4^	1 × 10^9^	Oxygen, corticosteroids, antibiotics	Survival	Wheezing at 1 year premature
Ursic et al. (16)	Slovenia, 2015	18 months	N/S	Respiratory distress	Hyperinflation and infiltrates of the right perihilar area, pulmonary edema	Blood, NPA, tracheal aspirate	PCR	–	No	Yes	Acute bronchiolitis with hypoxemia, diffuse hyperinflation, bilateral infiltrates, and possibly pulmonary edema	Yes	7.42 × 10^6^	8.27 × 10^6^	Oxygen, corticosteroids, antibiotics, dopamine	Death	Chronic respiratory insufficiency, premature
Jula et al. (10)	Finland, 2013	16 months	1/0	Rhinorrhea, cough, respiratory distress, tachypnea	Bilateral infiltrations and atelectasis of the upper right lobe	Blood, NPA	PCR/serology	HBoV IgM/IgG	low load HRV higher load HBoV1 in NPA	Yes	ARDS	Yes	3 × 10^3^	1.6 × 10^10^	Oxygen, corticosteroids, antibiotics	Survival	Severe bronchopulmonary dysplasia, premature
Calvo et al. (17)	Spain, 2008	1 months	1/0	Respiratory distress	Atelectasis	NPA	PCR	–	No	Yes	Respiratory failure	–	–	–	Oxygen, corticosteroids	Survival	Premature
		1 months	0/1	Wheezing, respiratory distress	Infiltrations	NPA	PCR	–	No	Yes	Respiratory failure	–	–	–	Oxygen, corticosteroids, antibiotics	Death	PDA/bronchopulmonary dysplasia, premature
Ziyade et al. (18)	Turkey, 2015	5 months	0/1	Fever, wheezing, diarrhea	–	NPA	PCR	–	No	Yes	Respiratory failure	–	–	–	Oxygen, Supplemental	Death	Premature

HBoV1, human bocavirus 1; ARDS, acute respiratory distress syndrome; NPA, nasopharyngeal aspirate; mNGS, metagenomics next-generation sequencing; CT, computed tomography; PCR, polymerase chain reaction; ECMO, extracorporeal membrane oxygenation; LRTI, lower respiratory tract infections; BHR/PSA, bronchial hyperresponsiveness/ paediatric status asthmaticus; NIN, ninein; HRV, human rhinovirus.

The risk factors for severe HBoV1 infections include underlying chronic conditions, such as congenital heart disease, chronic lung disease, premature birth, cancer, and immune deficiency (5). Of the 17 patients, 8 were premature infants, 6 had underlying pulmonary disease, 1 had patent ductus arteriosus (PDA), 2 had a history of wheezing, and 1 was an immunodeficient child. In terms of treatment, all the children were given oxygen: 8 cases were given antibiotic therapy, corticosteroids were given in 7 cases, mechanical ventilation was given in 14 cases, and ECMO in 2 cases. Four children died, and the remaining 13 survived. In this case, we performed qPCR detection of HBoV1 in the serum and NPA. The NGS and other PCR results showed that HBoV1 was the only detected pathogen. The viral load in the NPA was high, accompanied by viremia, suggesting that HBoV1 was the most likely cause of severe respiratory tract infection in this case. It is reported that the single detection of HBoV1 was more prevalent among children with a high viral load than those with a low viral load in severe acute cases (11). Christensen et al. (6) showed that mono-detection, high viral load, and viremia are associated with respiratory tract infection. The samples include blood, NPA, or tracheal intubation aspirates of the seven cases reviewed here for qPCR detection, and viremia was indicated in all seven critically ill patients. Unlike other markers (14, 22), the detection of HBoV1 DNA in the blood is more closely associated with the symptoms of the present infection. Therefore, blood testing is one of the important diagnostic methods for the study of HBoV1 (3, 5). It includes both PCR and serodiagnoses.

The results of whole exon sequencing showed a heterozygous variant in the *STAT1* gene: c.1127 + 10G > A, causing amino acid change splicing. According to the public database ClinVar, the clinical significance of this variant was considered to be uncertain significance. The family history was negative. STAT proteins are key transcription factors that regulate cellular responses to interferon (IFNs), cytokines, growth factors, and hormones and are associated with diseases related to regulating immune responses. The protein plays a vital role in immune responses to viral, fungal, and mycobacterium pathogens. The invasion of macrophages is the most common mechanism for infectious agents and *STAT1* is probably fundamental for the activation of the corresponding intracellular killing programs (23). Genetic variants in *STAT1* can lead to four different phenotypes. There are three outcomes of reduced or absent *STAT1* function (loss of function [LOF]) and one outcome of gained *STAT1* function (GOF). *STAT1* plays a crucial role in the cellular response to IFNA/IFNB (type I interferon) and IFNG (type III interferon). Autosomal dominant (AD) LOF STAT1 selectively affects the IFNG pathway but does not affect the IFNA/IFNB pathway and mainly leads to susceptibility to mycobacterial infections and no susceptibility to viral infections. AD LOF STAT1 has low penetrance, a mild clinical phenotype, and a good prognosis (24). However, AD GOF STAT1 with susceptibility to candida has a highly variable prognosis (25). Two patients with a heterozygous variant of *STAT1* have been reported to have an increased susceptibility to adult-onset herpes simplex encephalitis (HSE) without a history of other significant infections (24, 26). Autosomal recessive (AR) complete LOF STAT1 affects the IFNA/IFNB and IFNG pathways, leading to susceptibility to mycobacteria, Salmonella, and viruses, often leading to a severe course of the disease and fatal results (24). Patients with AR partial LOF STAT1 present with clinical insufficiency, and the severity of illness is variable (23). However, we have not collected samples from the parents of the child for genetic testing, so we cannot determine the source of the genetic variant. The clinical pathogenicity of this gene variant is uncertain.

In this case, we noted a continuous decline in hemoglobin and red blood cells, presenting as small cell hypochromic anemia. Still, the child had no signs of bleeding, considered to be iron-deficiency anemia or related to iron death. Jayaweera et al. (27) found a significant correlation between the risk of acute respiratory tract infections and iron deficiency anemia in children. Iron supplementation in blood played a critical protective role in recurrent acute respiratory tract infections and gastroenteritis in children. The child, in this case, had severe malnutrition, low oxygen-carrying capacity in pulmonary vessels and lung parenchyma, and low protective immunity against invading pathogens as well as a significant decrease in hemoglobin from day 10 after admission, suggesting a severe infection (28).

In this study, we used qPCR and mNGS for etiological detection, which further confirmed that HBoV1 was the only pathogen that could cause severe respiratory tract infection. Because the PCR method is often targeted at suspected pathogens, unknown or rare pathogenic microorganisms cannot be quickly identified, and certain limitations exist. The mNGS high-throughput sequencing of nucleic acids in clinical samples is then compared with the database analysis. The detection range is more extensive and suitable for diagnosing severe infections (29). The routine clinical use of mNGS is still under development. Assume that NGS is added to clinical and routine laboratory data. In that case, it may be combined with the PCR detection method for clinical diagnosis in the future, which has high clinical specificity and broad application prospects (30). Our study, using mNGS, provides further evidence that HBoV1 can cause severe acute respiratory tract infections in children without other viral and bacterial infections.

It is reported that at least two of the following five factors should be present for the diagnosis of an acute primary HBoV1 infection: high DNA load by qPCR (>10^6^ HBoV1 DNA copies/ml of NPA); HBoV1 mRNA in NPA; positive IgM; low IgG avidity; or a fourfold increase or more of IgG titre in paired serum samples (5). One of the limitations of this study is that we did not include the serological analysis of HBoV1. Second, as this study was a retrospective study, blood samples of the patient's parents were not collected; therefore, gene sequencing and variant analysis could not be performed for verification.

## Conclusion

In this case report, the clinical symptoms and signs of the child and the high viral load, viremia, and mNGS detection in the tracheal aspirate all supported that HBoV1 could cause a severe acute respiratory tract infection in children without other viral and bacterial infections. This case suggested that bocavirus can cause severe infection, especially in immunodeficiency conditions, and more vigilance is needed.

## Data Availability

The datasets presented in this study can be found in online repositories. The names of the repository/repositories and accession number(s) can be found in the article/Supplementary Material.
